# Supplementary material for: Anti-Inflammatory, Antimicrobial, Antioxidant and Photoprotective Investigation of Red Propolis Extract as Sunscreen Formulation in Polawax Cream
Source: Int J Mol Sci. 2023 Mar 7;24(6):5112. doi: 10.3390/ijms24065112 (PMC10049182; doi:10.3390/ijms24065112)
Supplement: Supplementary file 1 [file ijms-24-05112-s001.zip › ijms-2252117-supplementary.pdf]

SUPPLEMENTARY MATERIAL

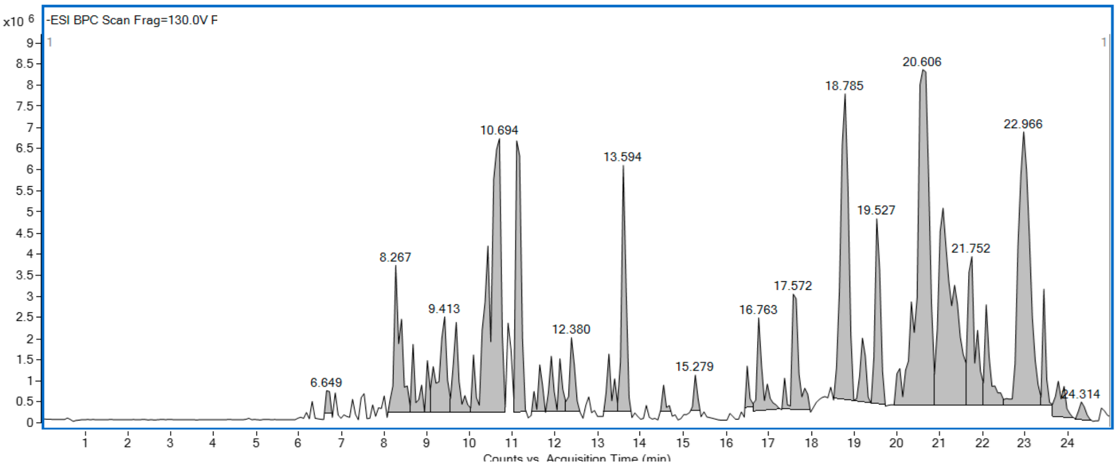

Figure S1. UPLC chromatogram of ethanolic red propolis extracts (EEPV) - Room Temperature.

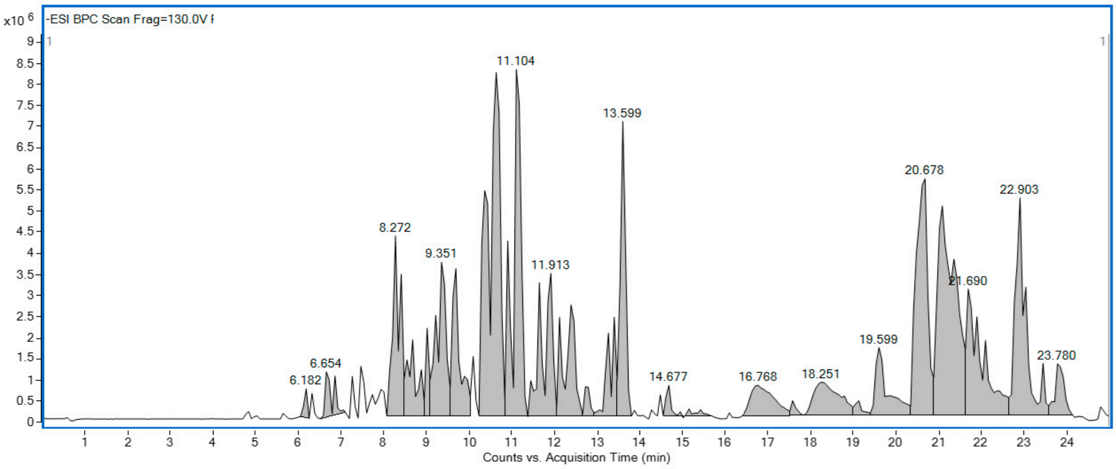

Figure S2. UPLC chromatogram of ethanolic red propolis extracts (EEPV) – Heated.

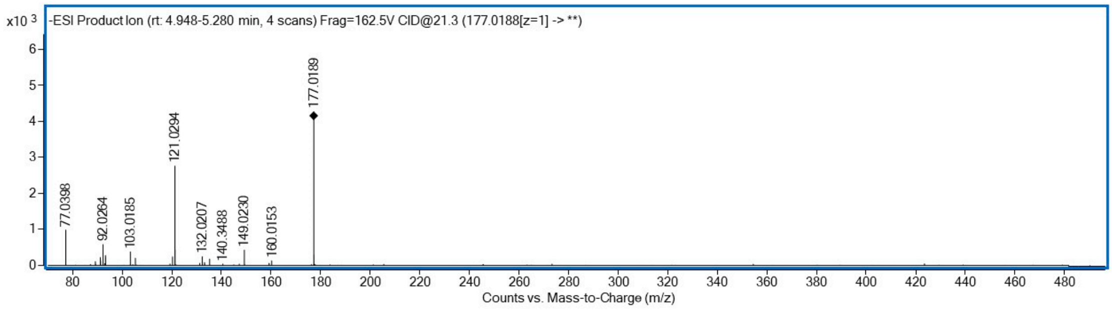

Figure S3. MS^2 spectra data of compound **1** $[M-H]^-$= 177.0188.

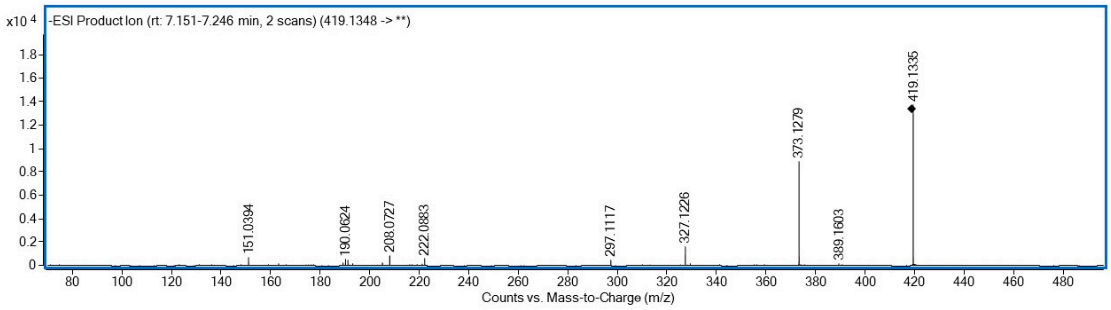

Figure S4. MS^2 spectra data of compound **2** $[M+FA-H]^-$= 419.1348.

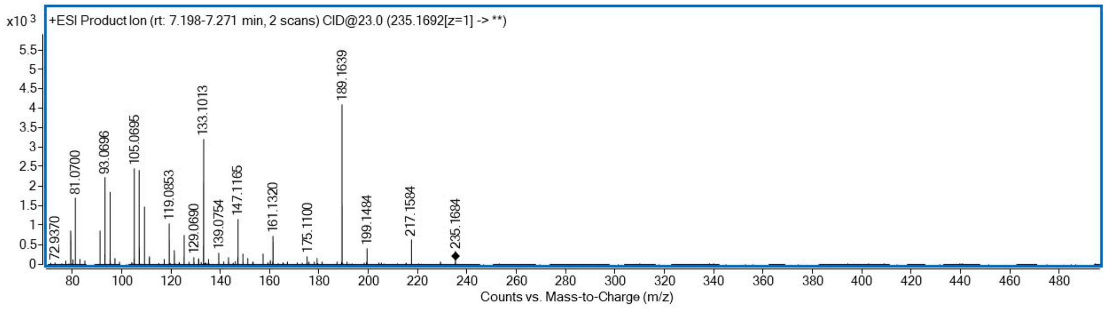

Figure S5. MS^2 spectra data of compound **3** $[M+H]^+$= 235.1692.

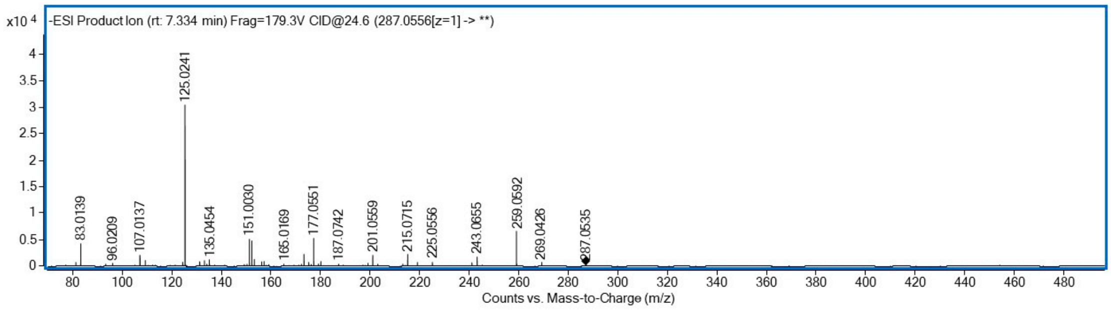

Figure S6. MS^2 spectra data of compound **4** $[M-H]^-$= 287.0556.

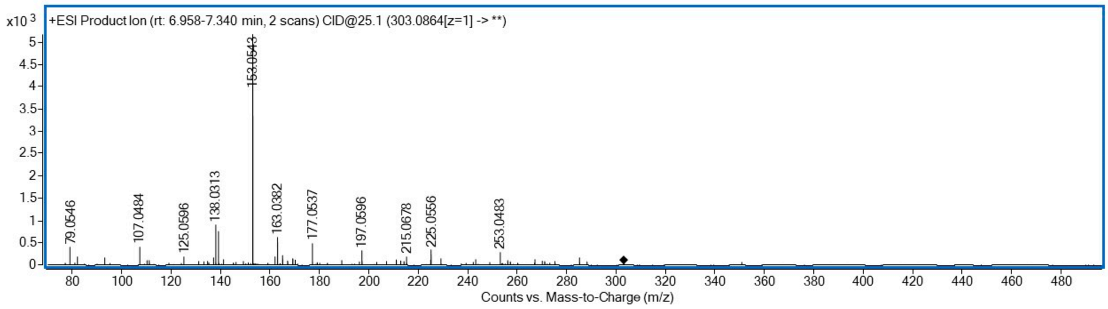

Figure S7. MS^2 spectra data of compound **5** $[M+H]^+$= 303.0864.

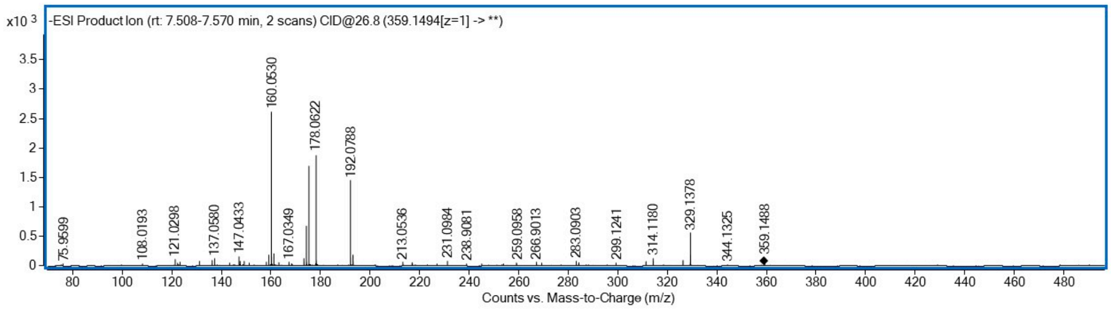

Figure S8. MS^2 spectra data of compound **6** $[M-H]^-$= 359.1494.

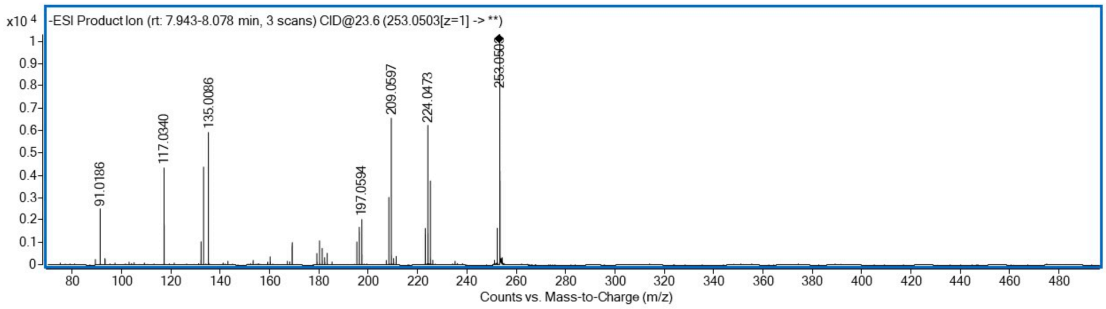

Figure S9. MS^2 spectra data of compound **7** $[M-H]^-$= 253.0503.

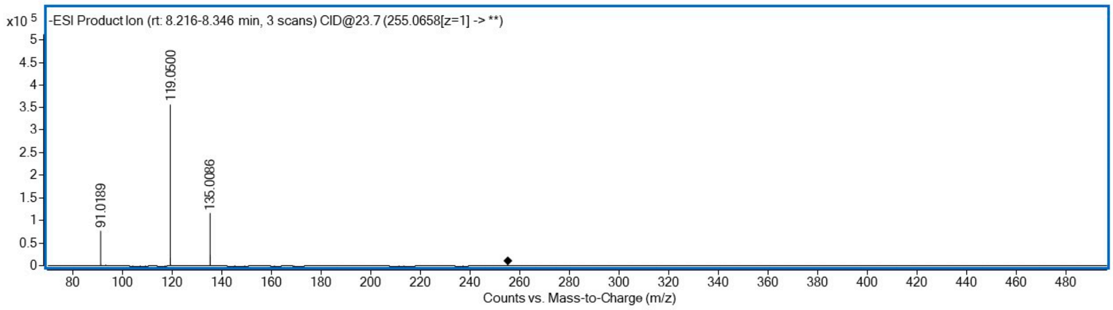

Figure S10. MS^2 spectra data of compound **8** $[M-H]^-$= 255.0658.

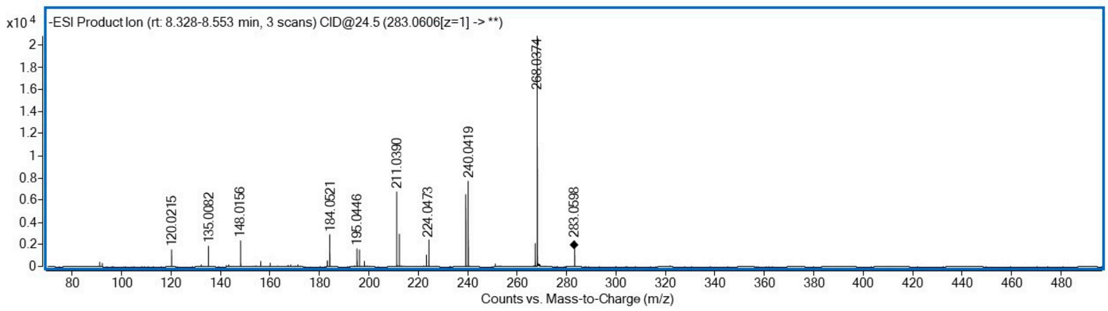

Figure S11. MS^2 spectra data of compound **9** $[M-H]^-$= 283.0606.

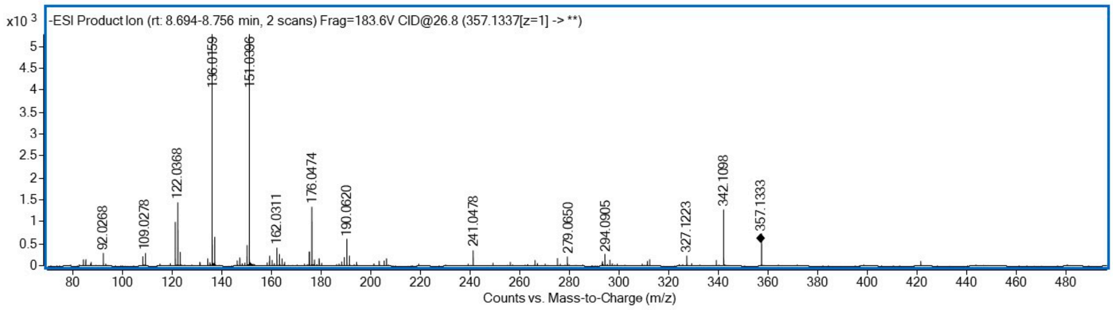

Figure S22. MS^2 spectra data of compound **10** $[M-H]^-$= 357.1337.

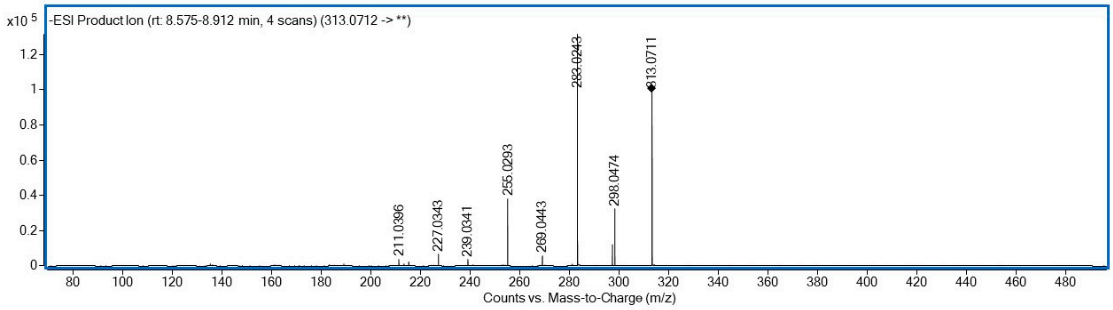

Figure S33. MS^2 spectra data of compound **11** $[M-H]^-$= 313.0712.

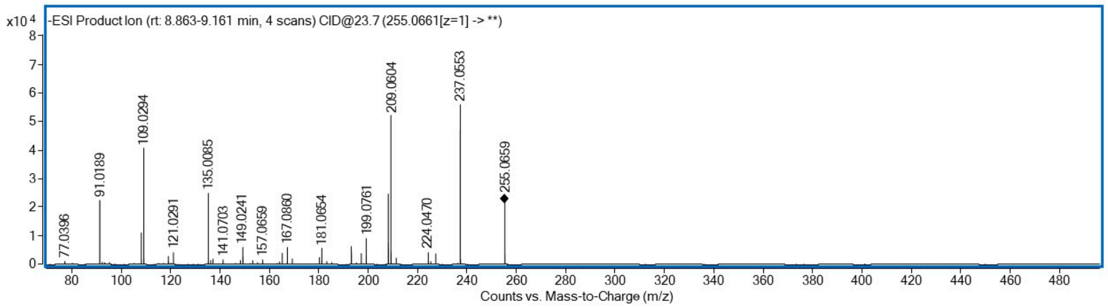

Figure S44. MS^2 spectra data of compound **12** $[M-H]^-$= 255.0661.

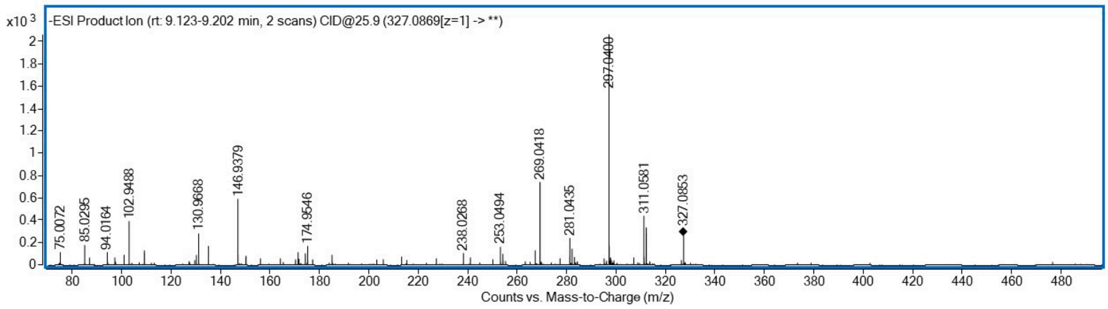

Figure S55. MS^2 spectra data of compound **13** $[M-H]^-$= 327.0869.

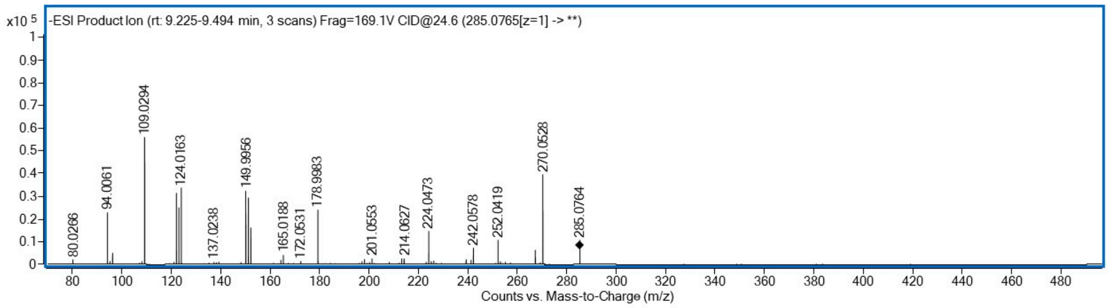

Figure S66. MS^2 spectra data of compound **14** $[M-H]^-$= 285.0765.

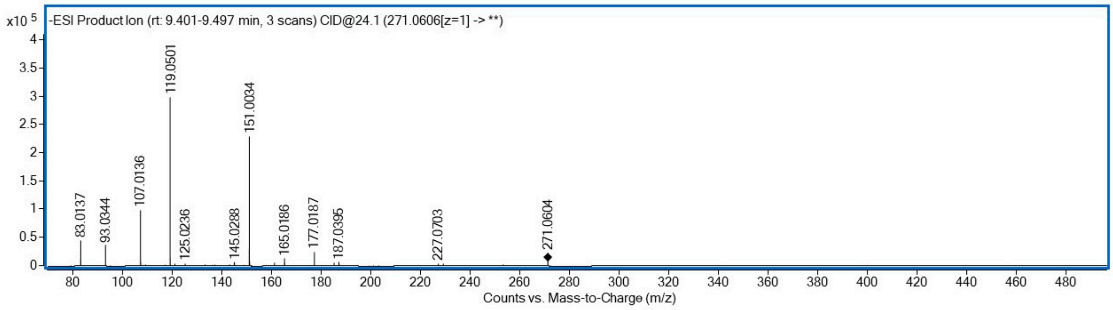

Figure S77. MS^2 spectra data of compound **15** $[M-H]^-$= 271.0606.

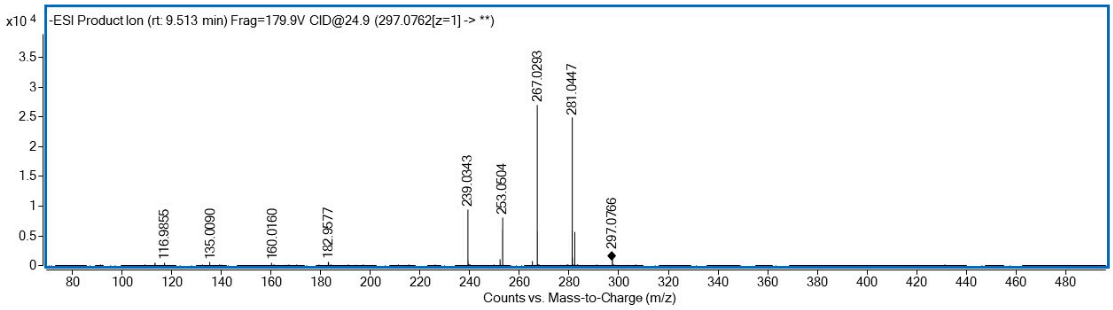

Figure S88. MS^2 spectra data of compound **16** $[M-H]^-$= 297.0762.

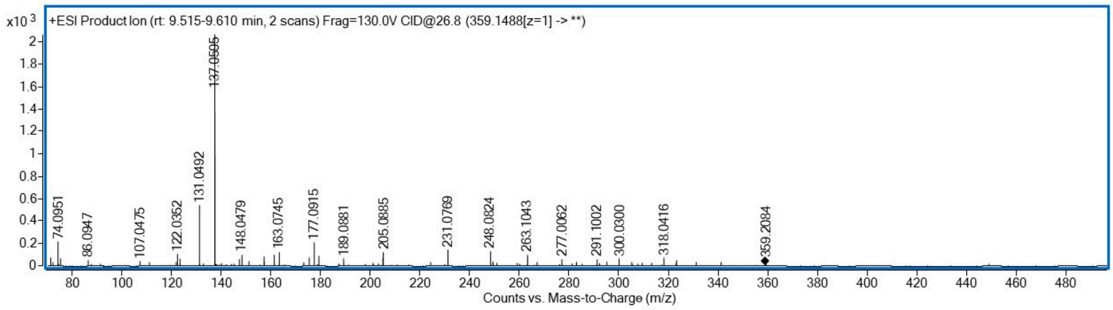

Figure S99. MS[2] spectra data of compound **17** [M+H]$^{+}$= 359.1488.

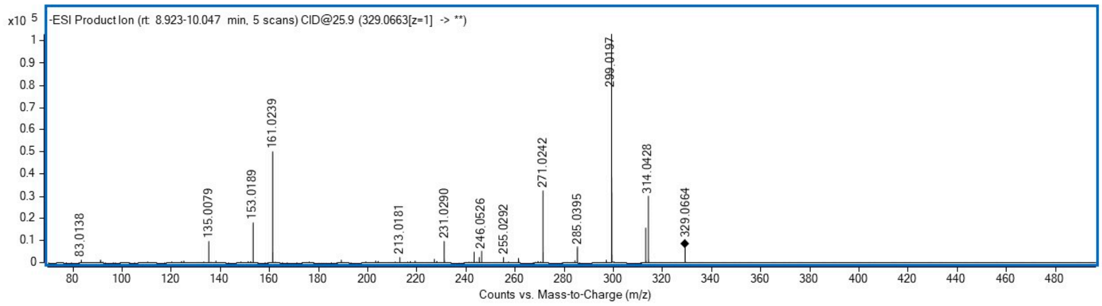

Figure S20. MS[2] spectra data of compound **18** [M-H]$^{-}$= 329.0663.

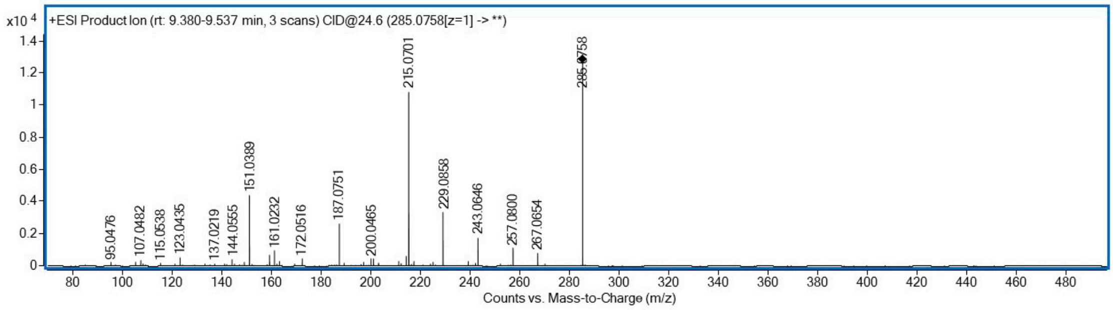

Figure S21. MS[2] spectra data of compound **19** [M+H]$^{+}$= 285.0758.

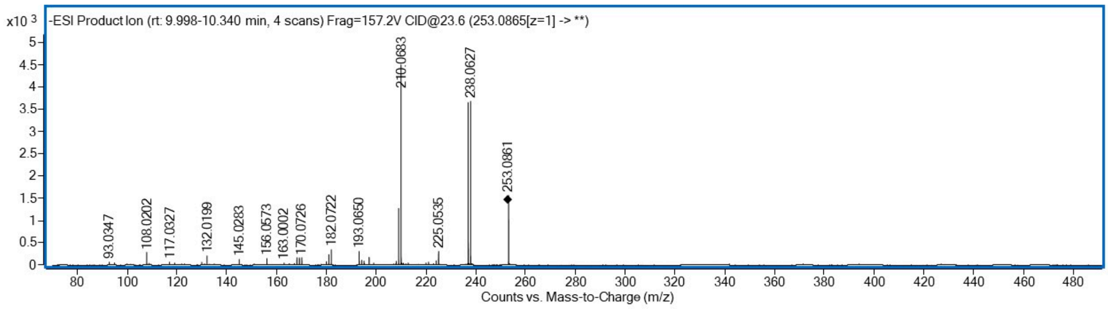

Figure S22. MS[2] spectra data of compound **20** [M-H]$^{-}$= 253.0865.

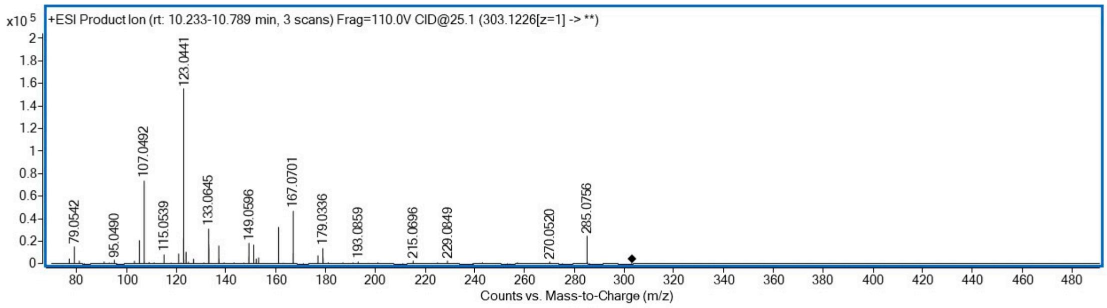

Figure S23. MS^2 spectra data of compound 2**1** $[M+H]^+$= 303.1226.

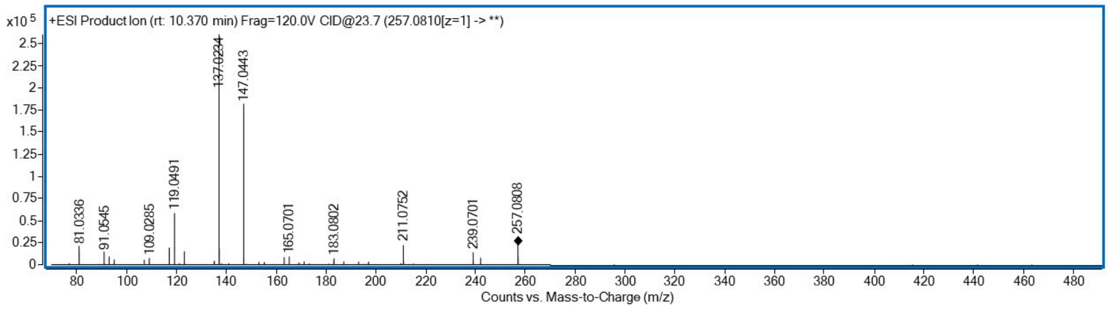

Figure S24. MS^2 spectra data of compound **22** $[M+H]^+$= 257.0810.

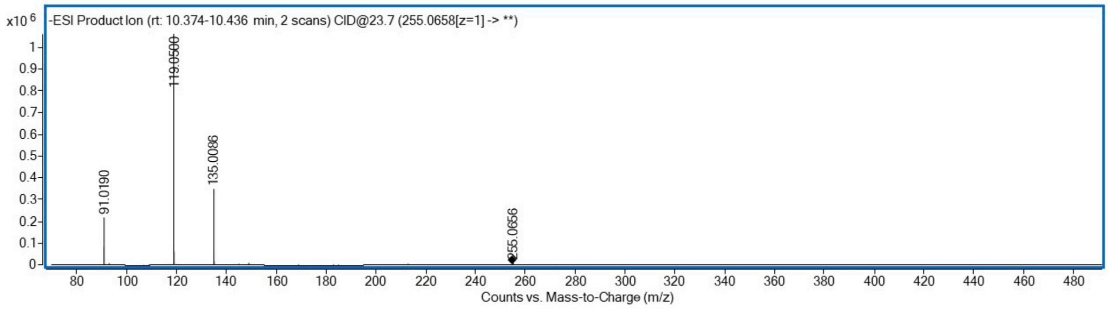

Figure S25. MS^2 spectra data of compound **23** $[M-H]^-$= 255.0658.

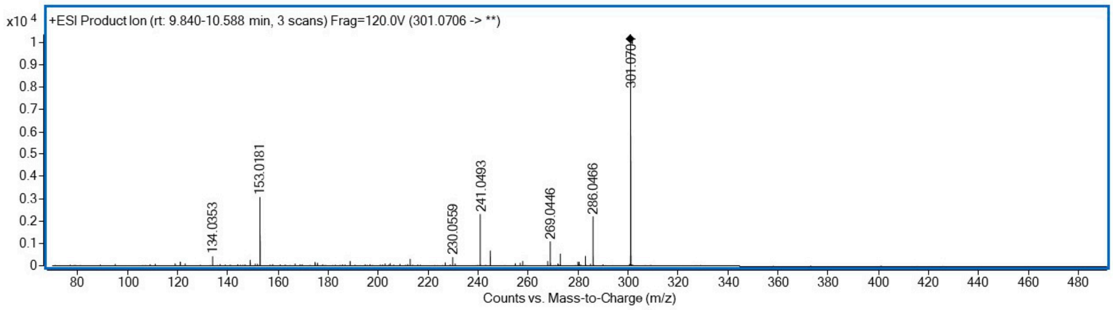

Figure S26. MS^2 spectra data of compound **24** $[M+H]^+$= 301.0706.

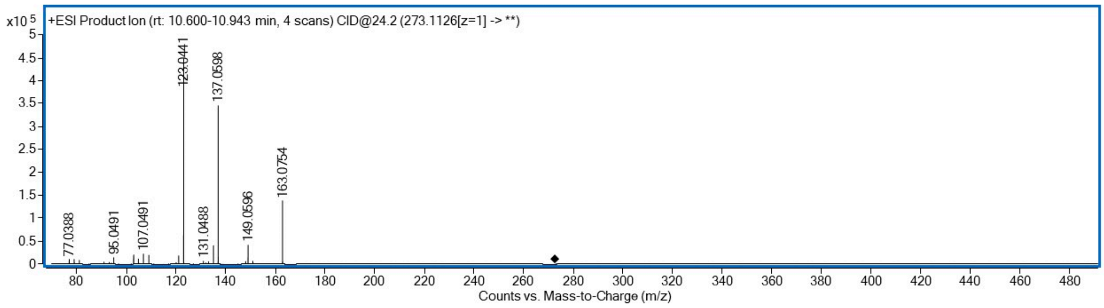

Figure S27. MS^2 spectra data of compound **25** $[M+H]^+$= 273.1126.

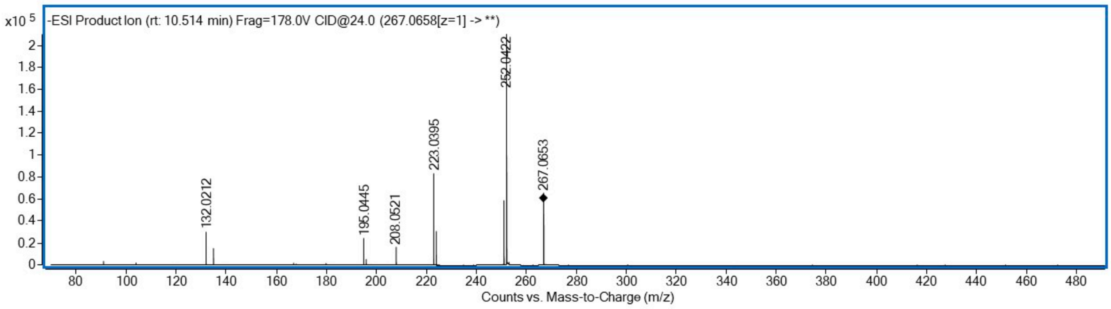

Figure S28. MS^2 spectra data of compound **26** $[M-H]^-$= 267.0658.

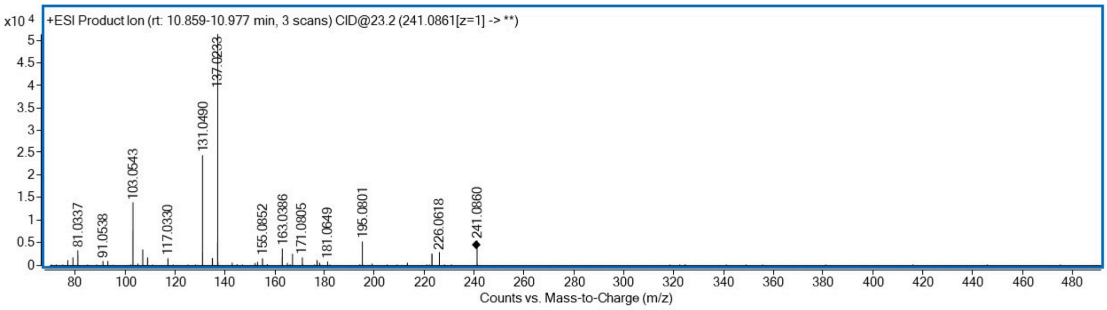

Figure S29. MS^2 spectra data of compound **27** $[M+H]^+$= 241.0861

.

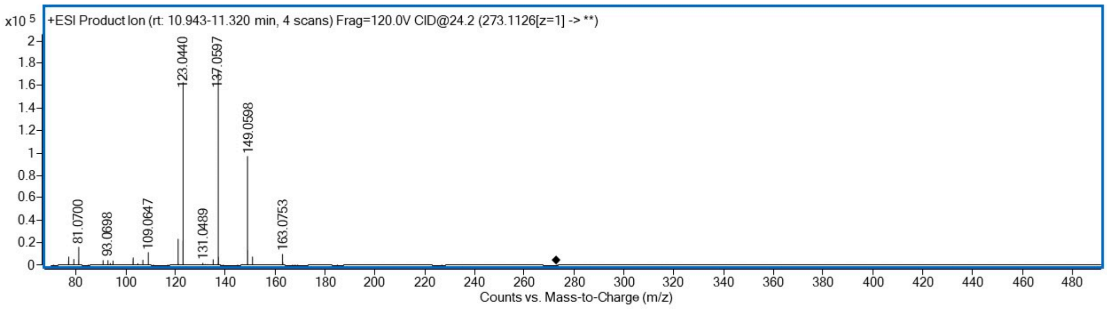

Figure S30. MS^2 spectra data of compound **28** $[M+H]^+$= 273.1126.

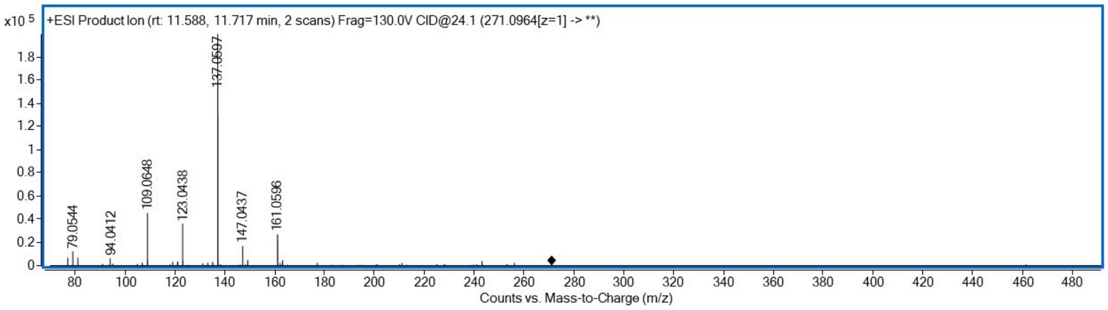

Figure S31. MS^2 spectra data of compound **29** $[M+H]^+$= 271.0964.

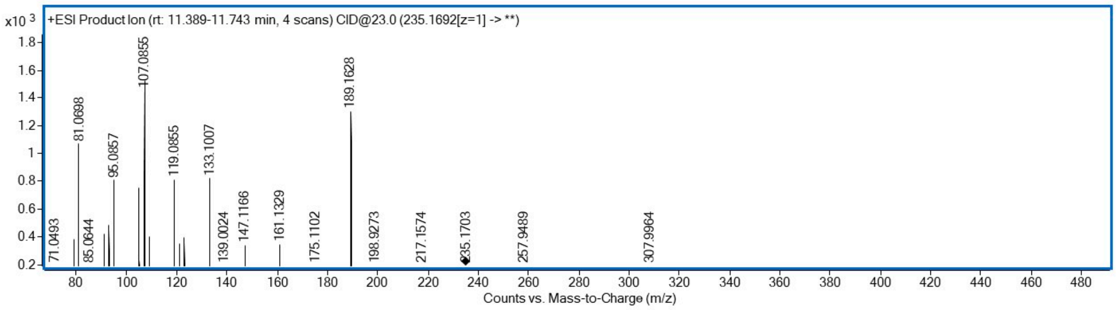

Figure S32. MS^2 spectra data of compound **30** $[M+H]^+$= 235.1692.

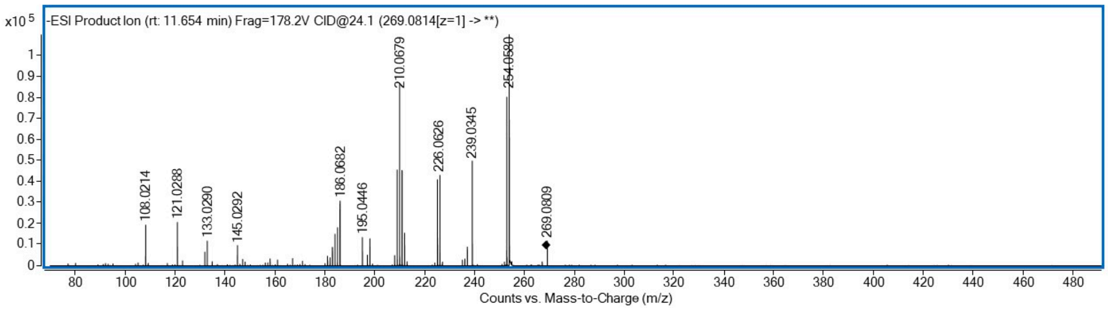

Figure S33. MS^2 spectra data of compound **31** $[M-H]^-$= 269.0814.

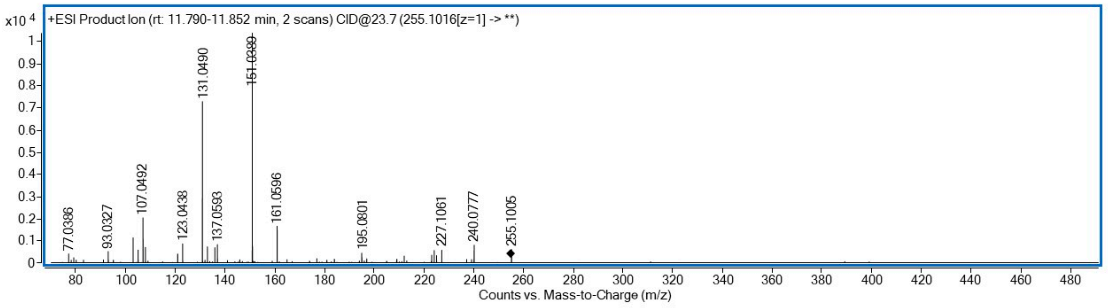

Figure S34. MS^2 spectra data of compound **32** $[M+H]^+$= 255.1016.

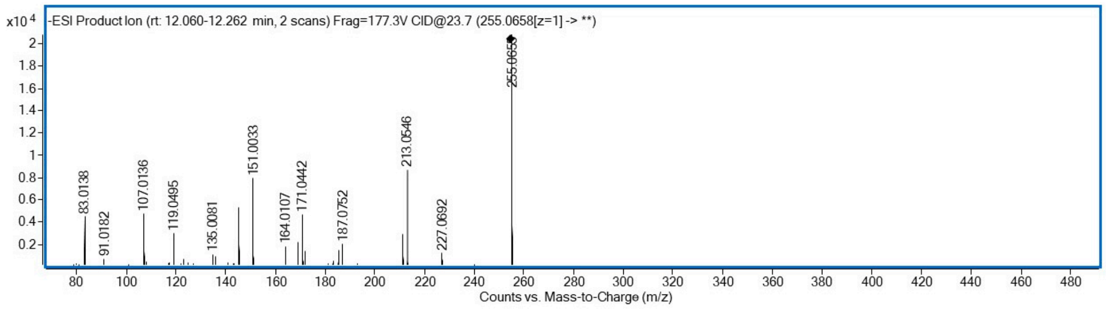

Figure S35. MS^2 spectra data of compound **33** $[M-H]^-$= 255.0658.

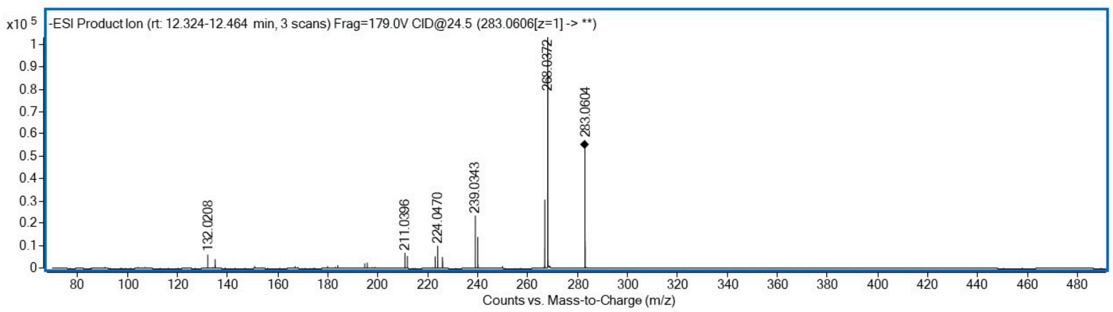

Figure S36. MS^2 spectra data of compound **34** $[M-H]^-$= 283.0606.

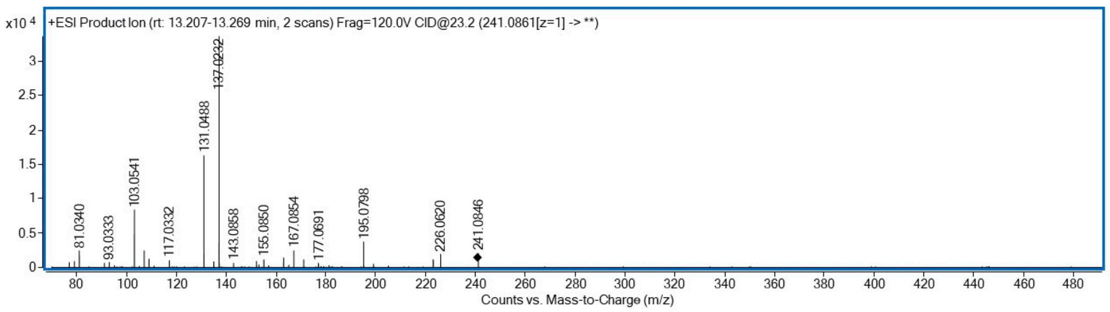

Figure S37. MS^2 spectra data of compound **35** $[M+H]^+$= 241.0861.

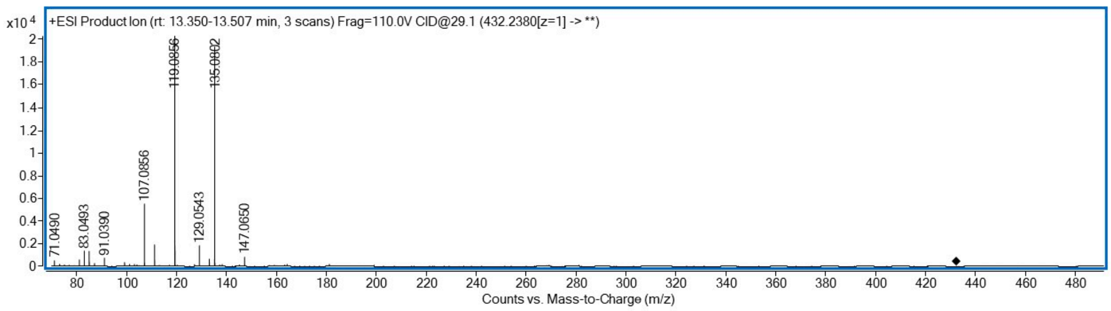

Figure S38. MS^2 spectra data of compound **36** $[M+ACN+H]^+$= 432.2380.

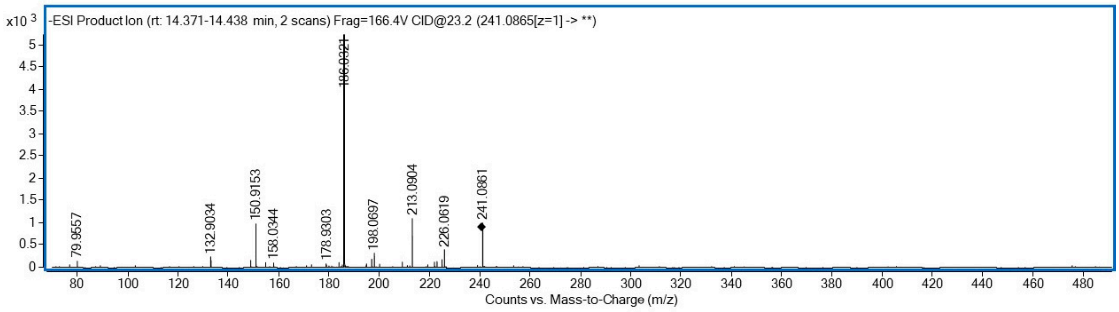

Figure S39. MS^2 spectra data of compound **37** $[M-H]^-$= 241.0865.

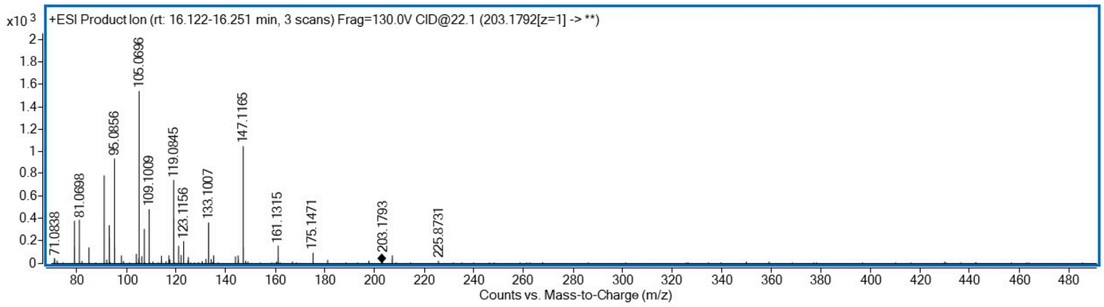

Figure S40. MS^2 spectra data of compound **38** $[M+H]^+$= 203.1792.

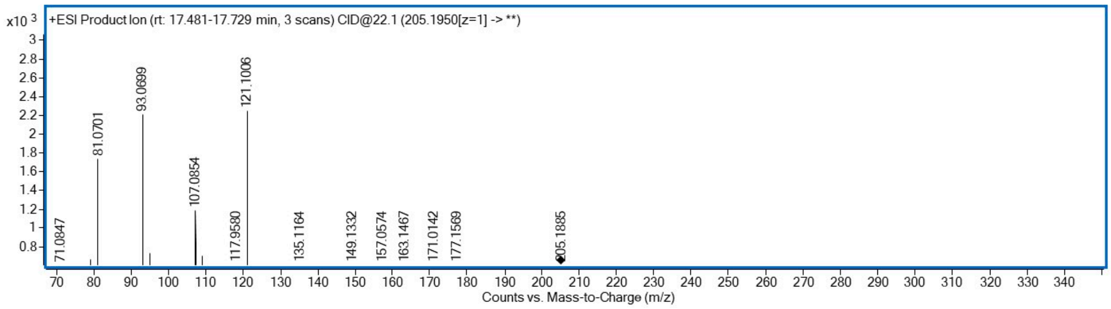

Figure S41. MS^2 spectra data of compound **39** $[M+H-H2O]^+$= 205.1950.

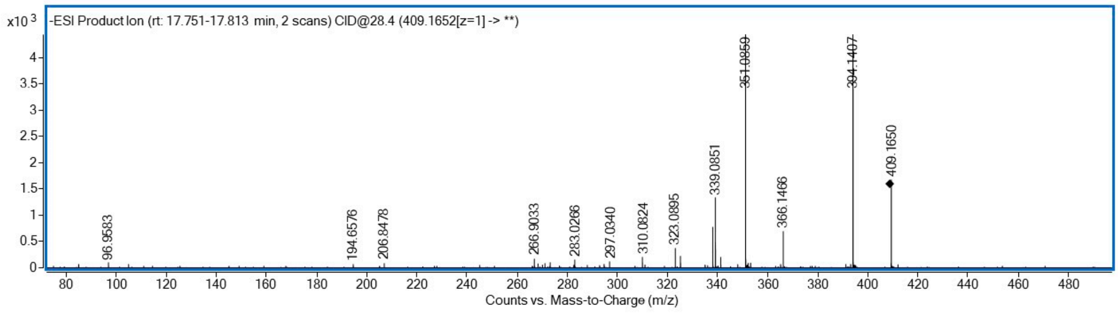

Figure S42. MS^2 spectra data of compound **40** $[M-H]^-$= 409.1652.

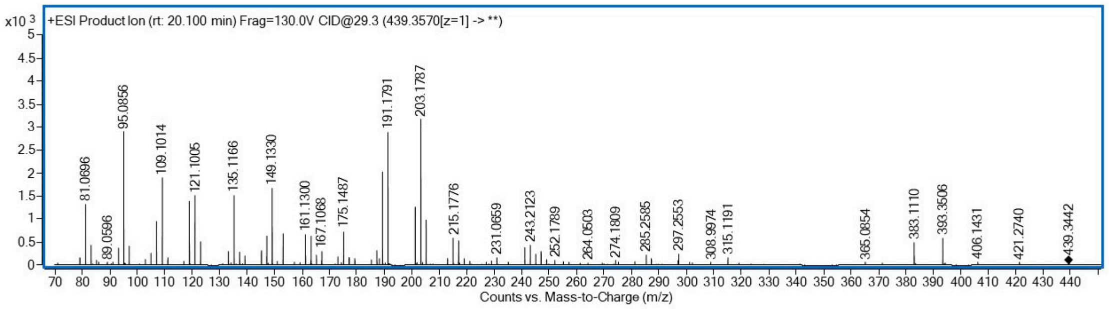

Figure S43. MS^2 spectra data of compound **41** $[M+H-H2O]^+$= 439.3570.

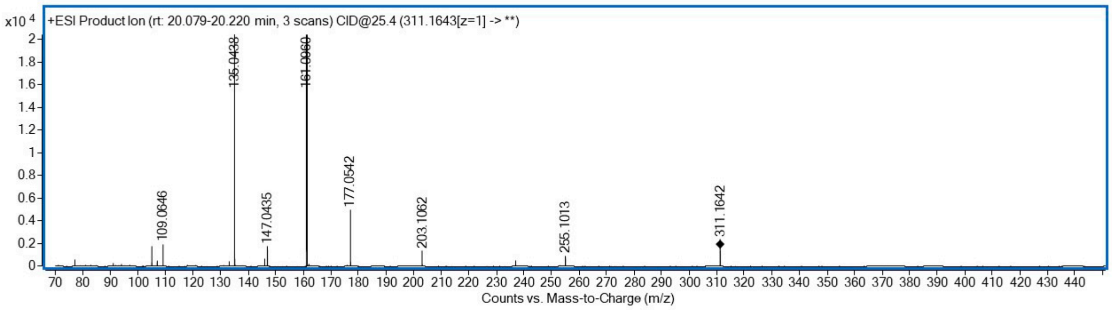

Figure S44. MS^2 spectra data of compound **42** $[M+H]^+$= 311.1643.

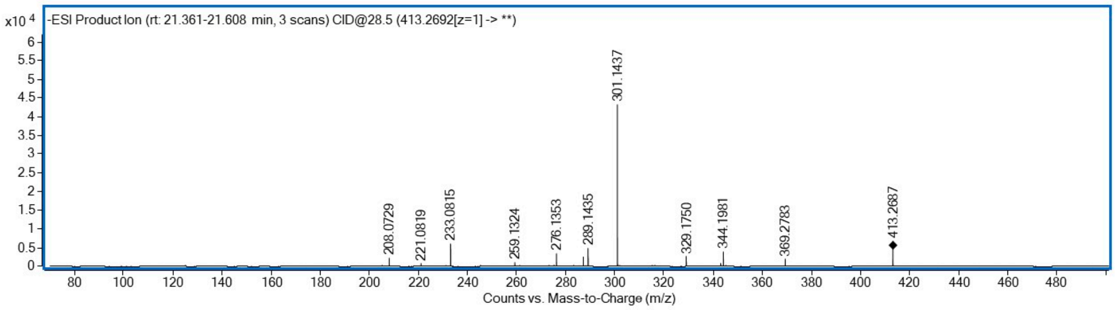

Figure S45. MS^2 spectra data of compound **43** $[M-H]^-$= 413.2692.
